# Refolding Techniques for Recovering Biologically Active Recombinant Proteins from Inclusion Bodies

**DOI:** 10.3390/biom4010235

**Published:** 2014-02-20

**Authors:** Hiroshi Yamaguchi, Masaya Miyazaki

**Affiliations:** 1 Liberal Arts Education Center, Aso Campus, Tokai University, Minamiaso, Kumamoto 869-1404, Japan; E-Mail: yamahiro@tokai-u.jp; 2 Measurement Solution Research Center, National Institute of Advanced Industrial Science and Technology, Tosu, Saga 841-0052, Japan; 3 Interdisciplinary Graduate School of Engineering Science, Kyushu University, Kasuga, Fukuoka 816-8580, Japan

**Keywords:** protein refolding, recombinant protein, inclusion body, denatured protein, chemical additive, microfluidic chip

## Abstract

Biologically active proteins are useful for studying the biological functions of genes and for the development of therapeutic drugs and biomaterials in a biotechnology industry. Overexpression of recombinant proteins in bacteria, such as *Escherichia coli*, often results in the formation of inclusion bodies, which are protein aggregates with non-native conformations. As inclusion bodies contain relatively pure and intact proteins, protein refolding is an important process to obtain active recombinant proteins from inclusion bodies. However, conventional refolding methods, such as dialysis and dilution, are time consuming and, often, recovered yields of active proteins are low, and a trial-and-error process is required to achieve success. Recently, several approaches have been reported to refold these aggregated proteins into an active form. The strategies largely aim at reducing protein aggregation during the refolding procedure. This review focuses on protein refolding techniques using chemical additives and laminar flow in microfluidic chips for the efficient recovery of active proteins from inclusion bodies.

## 1. Introduction

The expression of recombinant proteins is important for the study of the biological functions of genes, the development of therapeutic drugs [1], and for industrial processes [2]. There are several protein expression systems to produce the target proteins, such as bacteria, yeast, insect cells, mammalian cells, and cell-free systems. Expression in mammalian cells and insect cells produces biologically active proteins that contain post-translational modification(s), such as phosphorylation, acetylation, and glycosylation. However, expression using these systems gives low yields of the recombinant proteins, and the cost of these systems is generally expensive for industrial-scale protein production. Cell-free systems also give low yields of the recombinant proteins. Among the cell-free systems, the *Escherichia coli* (*E. coli*) overexpression system is the most convenient and frequently used to produce recombinant proteins [1,3]. 

Recombinant proteins often require the assistance of folding modulators, such as chaperone proteins, during expression in *E. coli*. In the overexpression system, the rate of protein aggregation is often much greater than that of proper folding and chaperone proteins are rapidly used up [4]. These protein aggregates are called inclusion bodies. As the target proteins are usually purified from soluble fractions in bacteria cell lysates, the formation of inclusion bodies is often a drawback in the use of *E. coli* expression systems [2]. Changing the growth conditions, such as growth temperature, concentration of inducer, and induction time, can often help in decreasing the formation of inclusion bodies (insoluble fraction of the proteins). Recently, it has been reported that recombinant proteins were overexpressed in the soluble form in the expression system by adding compatible solutes (chemical additives) in the expression medium [5]. Instead of forming inclusion bodies, the target protein could be expressed as a soluble protein in the presence of sorbitol, arginine, and trehalose in the expression medium [5]. It is expected that appropriate additives can suppress the formation of inclusion bodies and, thus, increase the solubility of target proteins in *E. coli* overexpression systems. 

As inclusion bodies contain relatively pure and intact recombinant proteins, several approaches have been reported to recover these aggregated forms as biologically active proteins. In a typical procedure, aggregated forms are denatured and dissolved with a high concentration of denaturant, such as urea, guanidinium chloride (GdnHCl), or ionic detergents, such as *N*-lauroylsarcosine. These chemical reagents are used to decrease the non-covalent interactions between protein molecules. In addition, dithiothreitol or 2-mercaptoethanol is added to reduce undesirable inter- and/or intra-molecular disulfide bonds. Refolding from denatured proteins (unfolded form) to active proteins (folded form) occurs by the removal of denaturant. Refolding efficiency (yield) of refolded protein can be estimated by biological activity, such as enzymatic activity. 

The procedure for the removal of the denaturant from denatured proteins is a key step in the efficient recovery of the proteins. Therefore, several approaches have been reported to refold an inactive protein into an active protein, such as size-exclusion chromatography [6], reversed micelle systems [7], zeolite absorbing systems [8], and the natural GroEL–GroES chaperone system [9]. These refolding methods, using chromatographic or non-chromatographic strategies, are introduced in recent reviews [10,11,12]. Although these methods work well for many inclusion body proteins and denatured model proteins, in most cases there is a significant amount of protein precipitation, resulting in a low recovery yield. Therefore, the protein refolding procedure is still performed with a series of trial-and-error refolding experiments. 

In this review, we focus on protein refolding methods using chemical additives and laminar flow in microfluidic chips for the efficient recovery of active proteins from inclusion bodies. In the first technique, the interesting features of chemical additives in dilution buffer are shown. The second method is a technique to remove denaturants from the denatured protein solution. With this technology, controllable diffusion by laminar flow in microchannels is used to control the denaturant concentration in a short period of time. Both strategies aim to inhibit the formation of protein aggregates during the refolding process. A brief overview of the refolding techniques discussed in this review is presented in Table 1. 

**Table 1:** Overview of refolding procedure (conditions) for the techniques in this review.

Technique	Recovery yield	Concentration of denatured protein	Time	Temperature
Dialysis	≤40% [13]	1–10 mg/mL	1–2 days	4 °C
Dilution *(chemical additive)*	≥80% [14]	1–10 mg/mL	1–2 days	4 °C
Microfluidic chip	≥70% [15]	≥250 μg/mL	≥10 min	≥25 °C

## 2. Conventional Refolding Methods to Remove Denaturants from Denatured Protein

### 2.1. Dialysis

**Figure 1:**
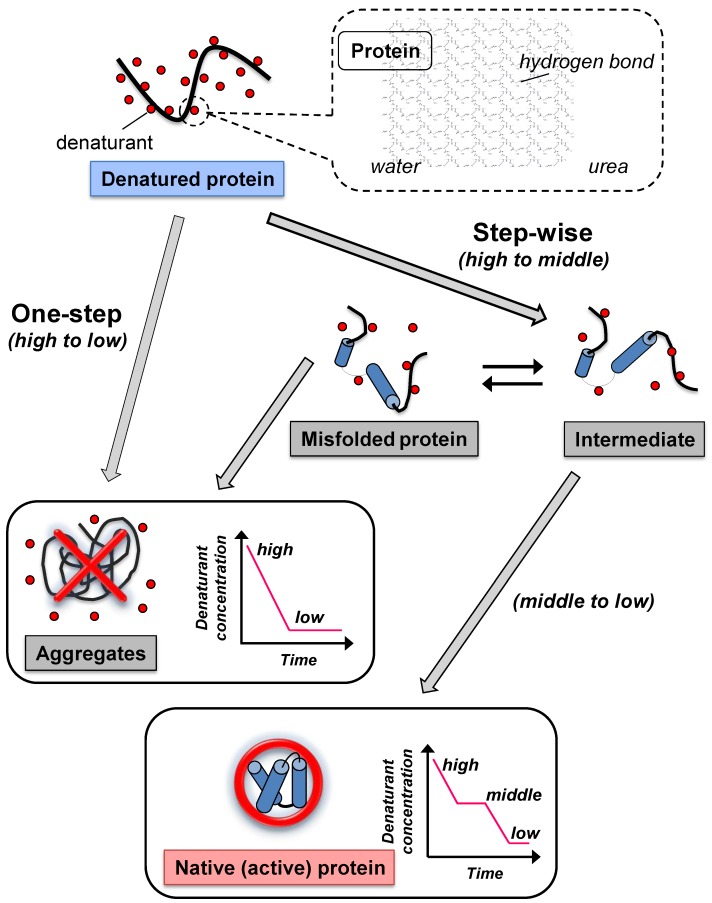
Schematic illustration of the two types of dialysis method for the removal of denaturants from denatured (solubilized) protein solutions. In one-step dialysis, the denaturant concentration around the protein rapidly decreases through diffusion, leading to aggregates. In step-wise dialysis, gradual removal of denaturant from the denatured proteins occurs.

In dialysis, the chemically denatured protein is refolded to sufficiently decrease the denaturant concentration and allow protein refolding. A schematic illustration of the dialysis methods is shown in Figure 1. One-step dialysis (high denaturant concentration with respect to the refolding buffer) is a simple method. The protein concentrations are almost constant during the refolding procedure. As the concentration of denaturant decreases when increasing dialyzing time, the rate of refolding to the native (active) structure increases. However, the rate of misfolding and protein aggregation will also increase, possibly due to contact between exposed hydrophobic surfaces [2,16]. This suggests that the rapid decrease in denaturant concentration initiates the reformation of aggregates, as observed in the dilution method (Section 2.2). 

To solve this problem, step-wise dialysis has been used. In step-wise dialysis, the denatured proteins are first brought to equilibrium with a high denaturant concentration, then, the concentration is decreased and brought to equilibrium at a medium concentration, and, then, further decreased and brought to equilibrium at a low concentration. This suggests that gradual removal of denaturant from the denatured proteins can achieve high refolding efficiency [17,18]. Although the step-wise dialysis method may give the refolded (active) proteins, it is a time-consuming procedure (multi-day). In addition, at medium denaturant concentrations the proteins often take the inactive form during the refolding process due to the reformation of aggregates and the presence of other misfolded species (Figure 1) [3,17]. Recent studies have found that protein aggregation predominantly occurs at medium concentrations of denaturant [19,20], suggesting that the refolding procedure, in a short period of time, may reduce the formation of protein aggregation and result in efficient protein refolding.

### 2.2. Dilution

The dilution method is also widely used for the removal of denaturant because the procedure is simple. The denatured proteins are merely directly diluted by a hundred to a thousand times with a refolding buffer that does not contain denaturants. In the dilution method, the protein concentrations are also decreased. Aggregation is known to be a function of the protein concentration. Therefore, a low concentration of the protein should be suitable to avoid intermolecular aggregation during the procedure. However, the diffusion coefficient of the denaturant is much larger than that of proteins, indicating that the denaturant diffuses faster than the protein at the initial point of the dilution method. Under such a removal of denaturants, denatured proteins immediately aggregate, similar to in one-step dialysis. In addition, this method requires a large volume of buffer to dilute the denaturant that does not disturb refolding. Moreover, difficulties are encountered in uniform mixing in a large volume, wherein reformation of aggregates occurs. 

## 3. Protein Refolding Using Chemical Additives

**Figure 2:**
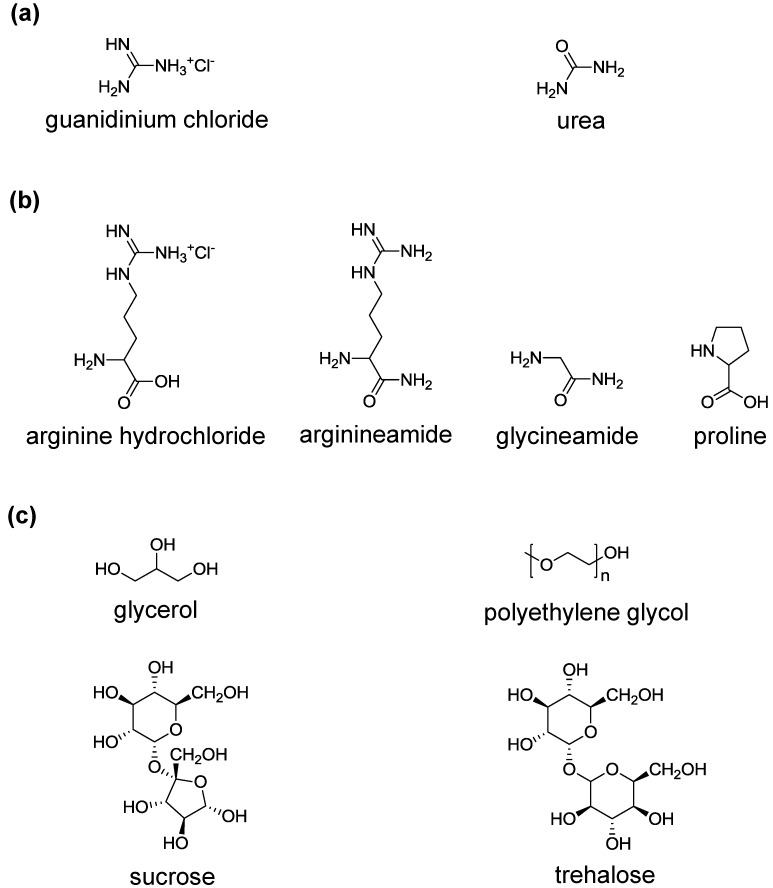
Typical chemical additives used for protein refolding. (**a**) Denaturants; (**b**) Inhibitors; (**c**) Stabilizers.

Protein refolding from denatured proteins is influenced by several factors, including solubility of protein, removal of denaturant, and assistance of refolding by co-solute or additives. As described in Section 2, formation of aggregated proteins often occurs during refolding by dialysis and dilution. The addition of small chemical molecules has been frequently used to prevent protein aggregation. The chemical additives can be classified into three types: denaturants, protein stabilizers, and protein aggregation inhibitors. Typical structures of the types of chemical additives are shown in Figure 2.

Urea and GdnHCl are typical protein destabilizers (denaturants). At high concentrations, they mostly denature proteins by the chaotropic effect. In contrast, it is known that, at low denaturant concentrations, they tend to stabilize the structure of the target protein, leading to inhibition of protein aggregation during refolding [21,22]. Although the mechanism remains unclear, at low concentrations the aggregation rate markedly decreases compared with the refolding rate. (NH_4_)_2_SO_4_ is a protein stabilizer that can stabilize the protein structure at low concentration through electrostatic interactions, which changes the solubility of the native (folded) structure protein. However, protein destabilizers often accelerate protein aggregation. Some amino acids are classified as protein aggregation inhibitors, including arginine and its derivatives. Recently, this type of chemical additive has been widely used for the refolding process to increase refolding yields by decreasing aggregation. 

Due to these interesting features of protein refolding, chemical additives are often added to the refolding buffer in the dialysis and dilution methods to decrease the degree of aggregated and/or misfolded proteins. Thus, many additives have been developed and used to improve the recovery yields of the active proteins [23]. In many cases, the inclusion body proteins have been successfully refolded. In this section, we introduce inhibitors and stabilizers (Figure 2) and discuss the application of the additives, and, then, discuss artificial chaperone-assisted refolding.

### 3.1. Amino Acids

The addition of arginine hydrochloride (ArgHCl) or arginine (Arg) to the refolding buffer has been reported to have interesting effects during the refolding process, such as improving the solubility of proteins and inhibiting protein aggregation. Thus, Arg has been studied as an additive for refolding of denatured model proteins [14,24,25] and has been applied for the refolding of a variety of proteins from inclusion bodies, such as casein kinase II [26], gamma interferon [27], p53 tumor suppressor protein [28], and interleukin-21 [29]. 

**Figure 3:**
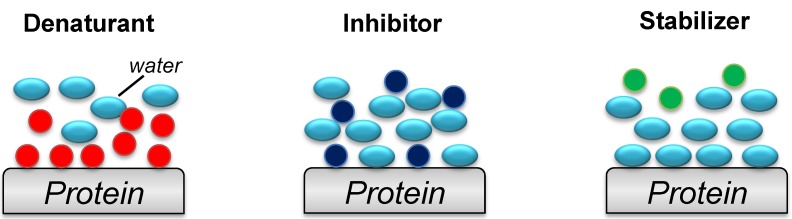
Models of the interactions between the surface of the protein with water and chemical additives. The additives and molecular water are represented by colored circles and ellipses, respectively: denaturant (strong chaotropic reagent), red; inhibitor (moderate chaotropic reagent), dark blue; stabilizer (osmotic reagent), green; and water, light blue.

Despite its effectiveness, the molecular mechanism of Arg is still unclear. ArgHCl has a guanidinium group (Gdn) at the distal end of its side chain, which is similar to GdnHCl (Figure 2). Gdn strongly interacts with hydrophobic amino acids in hen egg-white lysozyme [30] and acts as a strong chaotropic reagent. The crystal structure of the lysozyme–Arg complex showed that Arg bound at sites different from those of Gdn, and mildly interact with nearby hydrophobic amino acid of the protein surface [31]. These interactions included electrostatic, hydrophobic, and cation–π interactions. Compared with Gdn, the amino and carboxyl groups of Arg form weaker hydrogen bonds with the denatured protein and water (Figure 3). Thus, Arg might act as an aggregation inhibitor due to its moderate binding to proteins [32]. 

In addition to Arg, other amino acids have been reported to be moderate chaotropic agents. Arginineamide and glycineamide have been reported to be aggregation inhibitors for protein refolding and increase the refolding yields of the proteins better than Arg [32,33,34]. Although their moderate chaotropic effects are unclear, the crystal structure of the lysozyme–glycine amide complex indicated that glycine amide binds to the protein surface near hydrophobic residues, such as aromatic residues, decreased the amount of bound water, and increased the mobility of the protein [34]. In addition, the binding sites of glycineamide were different from those of Arg, and almost all of the bound glycine amide only formed hydrogen bounds between the carboxyl amide group and surface residues of the protein and hydrated water [34]. These results support that the different binding sites of glycine amide and Arg probably lead to their different inhibitory abilities. 

Proline has also been reported to enable proteins refolding to their native (active) conformation. It was proposed that proline inhibits protein aggregation by binding to the folding intermediate(s) and trapping the folding intermediate(s) in the supramolecular assembly with proline [35]. 

### 3.2. Glycerol, Polyethylene Glycol and Sugars

Glycerol acts as a protein stabilizer (Figure 2 and Figure 3) by enhancing the hydrophobic interactions as a consequence of an increase in the solvent ordering around the proteins. Increasing the glycerol concentration increases the stability of proteins even at high protein concentrations [36,37]. Similarly, polyethylene glycol [38,39] and sugars [40,41] have been reported to act as stabilizers of protein structures. For example, polyethylene glycol has been successfully used for refolding of insulin-like growth factor [42] and interferon [43]. During the refolding process, these additives bind to the intermediate in the folding pathway of the protein or interact with the hydrophobic side chains of the denatured protein. It has been suggested that these additives create an ideal environment where the refolding rate increases while the aggregation rate decreases [36,37,38,39,40,41]. Although such stabilizers increase the refolding yields, protein aggregation simultaneously occurs. Therefore, these types of additives have always been used in combination with an aggregation inhibitor such as Arg [37,42]. 

### 3.3. Cyclodextrins

Cyclodextrins have been reported to inhibit protein aggregation during the refolding process [44,45,46]. Cyclodextrins have an amphipathic structure. The hydrophobic cavities and hydrophilic groups (mainly hydroxyl group) can interact with proteins by weak interactions, such as hydrogen bonds, van der Waals interactions, and hydrophobic interactions, and probably have a similar moderate chaotropic effect to ArgHCl. 

In addition to their effect as a protein aggregation inhibitor, cyclodextrins have been used for other refolding methods. In host–guest chemistry, it is well known that cyclodextrins, such as β-cyclodextrin, can capture chemical compounds in their central hydrophobic cavities. Based on this property, cyclodextrins have been used as stripping agents for the removal of detergents from denatured protein by surfactants. This refolding method is called artificial chaperone-assisted refolding [47,48]. The method is based on chaperonin proteins in bacteria. Bacterial GroEL and GroES proteins (chaperonin) are molecular chaperones for protein refolding *in vivo*. In this system, misfolded protein is captured in the hydrophobic central cavity by the GroEL tetradecamer. When the GroES heptamer caps the cavity, protein refolding occurs in an ATP-dependent manner. The artificial chaperone-assisted refolding method mimics the GroEL-GroES system [47,48]. The refolding involves two steps. In the first step, the denatured protein is diluted with a buffer containing detergents that prevent protein aggregation. In the second step, the protein–detergent complex solution is diluted with a buffer containing cyclodextrins that strip detergent from the complex. Various cyclodextrin derivatives and detergents have been synthesized for effective protein refolding [49]. A large variety of proteins have been refolded by the artificial chaperone-assisted refolding methods [47,48,49]. 

## 4. Decreasing Denaturant Concentration by Laminar Flow in Microfluidic Chips

It has been suggested that the refolding procedure in a short period of time may reduce the formation of protein aggregates and achieve efficient protein refolding [19,20]. However, it is difficult to efficiently refold proteins in a short time using the conventional methods discussed in Section 2 and Section 3. Although the chemical additives described in Section 3 are helpful to inhibit protein aggregation, the procedure usually requires multiple days. To overcome this difficulty, we have proposed the microfluidics approach as a rapid and simple refolding method [15]. 

Microfluidic reaction systems are widely studied and used in chemistry and biotechnology fields [50,51,52,53]. The laminar flow in microchannels can be used to create a well-defined and predictable interfacial region among the streams. Additionally, diffusion mass transfer is enhanced in the microchannel compared with in the macrochannel. These characteristics inspired us to control the gradual removal of denaturants from chemically denatured proteins in the laminar flow. Previous reported microfluidic chips for refolding were designed to study the initial folding events during rapid mixing of the denatured protein solution and refolding buffer through either turbulent flow [54,55] or diffusion [56,57]. In these kinetic studies, easy-to-fold proteins where the measured protein folding occurs within 100 μs [54], such as cytochrome c, were used. In contrast, difficult-to-fold proteins, which often form inclusion bodies, aggregate in the microchannel by rapid mixing [58] due to rapid removal of denaturant from the denatured proteins. It suggests that microfluidic chips with rapid mixing are not applicable for refolding of difficult-to-fold proteins. 

### 4.1. Design of Microfluidic Chips

Gradual decrease of denaturant concentration from the denatured protein within a short period of time may lead to efficient protein refolding. The concept of protein refolding using microfluidic chips is based on controllable diffusion of the denaturant from the denatured protein stream to the diluting buffer in laminar flow. A similar concept of controllable diffusion of reagents by hydrodynamic flow has been applied for the synthesis of polymeric nanoparticles [59] and for rapid protein concentration analysis [60].

**Figure 4:**
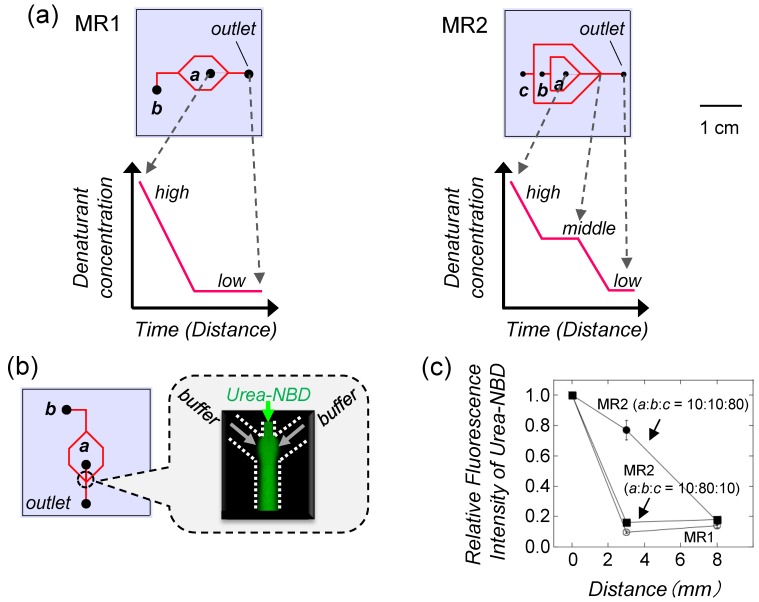
Microfluidic chip used for protein refolding [15]. (**a**) Designed microfluidic chips. In MR1, the denaturant concentration around the protein rapidly decreases because of diffusion, which is expected to have a similar mechanism to one-step dialysis and dilution. In MR2, the denaturant concentration shows a step-wise decrease, which is a similar mechanism to step-wise dialysis. The denatured protein was injected into channel *a*. The diluting buffer was injected into channels *b* and *c*; (**b**) Confocal fluorescence microscope image at the junction in MR1 showing laminar flow of the urea stream through the diluting buffer streams. The focused urea stream contains *N*-(4-nitrobenzo-2-oxa-1,3-diazolyl)amine (NBD) as an indicator; (**c**) Relative fluorescence intensities of NBD in the urea stream as a function of the distance from the inlet (position *a*). Flow rates (μL/min) are shown in the graph.

In laminar flow in the designed chips (Figure 4a), the fluid stream to be mixed flows along the central stream (denatured protein) and encounters two buffer streams at junction *a*. In general, the mean square displacement (<*x*^2^>) of molecules in solution is proportional to mixing time *t*:

<*x*^2^> = 2*Dt* (1)

where *D* is the diffusion coefficient and is of the order of 10^−7^ cm^2^/s for proteins (0.5 – 8.7 × 10^−7^ cm^2^/s) [61] and of the order of 10^−5^ cm^2^/s for small molecules like urea (1.4 × 10^−5^ cm^2^/s) [62], indicating that denaturants diffuse two orders of magnitude faster than proteins. In addition, at low Reynolds number (*Re* < 20 under our experimental conditions), the central stream of denatured protein is squeezed into a narrow stream between the two adjacent buffer streams. The width of the focused stream depends on the flow rate of the diluting buffer [59,63]:

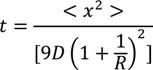
(2)
where *R* is the ratio of flow rate of the denatured protein to the flow rate of the refolding buffer [59]. The denaturant in the central stream of the denatured protein then enables mixing with the buffer by diffusion and the denaturant concentration decreases, meaning that the ratio of flow rates of the refolding buffer can control the denaturant concentration in the microchannel. 

To refold the protein, the denaturants in the denatured proteins should be diluted at least 10-fold [15]. However, it is expected that a direct 10-fold dilution at the junction in the microchannel may induce misfolding or aggregation [15]. Although varying the flow rate of the diluting buffer can control the distribution of denaturant concentration (Equation 2), a chip with only a single junction cannot generate the gradual decrease of denaturant required for efficient protein refolding. It is expected that better control of the concentration of the denaturant will be achieved by increasing the number of junctions (buffer streams) compared with the chip with only one junction. Therefore, a chip with multiple junctions was designed to generate the gradual decrease of denaturant concentration. Based on these ideas, we designed two types of microfluidic chips: MR1 and MR2 (Figure 4a). The denatured proteins are directly diluted by the buffer in MR1 (one junction), which is expected to have a similar mechanism to one-step dialysis or dilution. On the other hand, MR2 has two junctions that can control the different flow rate ratios of the buffer streams (channels *b* and *c*). 

To confirm whether the laminar flow in the designed chips can control the distribution of denaturant concentration, a urea stream in the microchannel was studied by confocal fluorescence microscopy (Figure 4b). As common denaturants, such as urea, do not have a fluorescent property, hydrophilic NBD, which has a similar molecular size and diffusion coefficient to urea, was added to the urea stream as a fluorophore. It is possible that the fluorescence intensity of NBD may be affected by urea concentration. The focus of the microscope was adjusted at position *a* (inlet) in Figure 4a. NBD diluted by the buffer at the junction shows a decrease in fluorescence intensity compared with that at the inlet, indicating that the urea concentration decreased (Figure 4c). The results indicate that the laminar flow in the designed chips can control the distribution of denaturant concentration, as expected [15].

### 4.2. Protein Refolding Using Microfluidic Chips

To test the performance of the designed microfluidic chips, the refolding of citrate synthase (CS) was tested [15]. CS is known to have low refolding yield using dialysis and dilution [13]. Therefore, CS has been used as a test case for refolding strategies [47,49]. In addition, CS is a dimeric protein composed of two identical subunits, suggesting that CS is a good model protein to study, not only the secondary and tertiary structures, but also the quaternary structure. 

**Figure 5:**
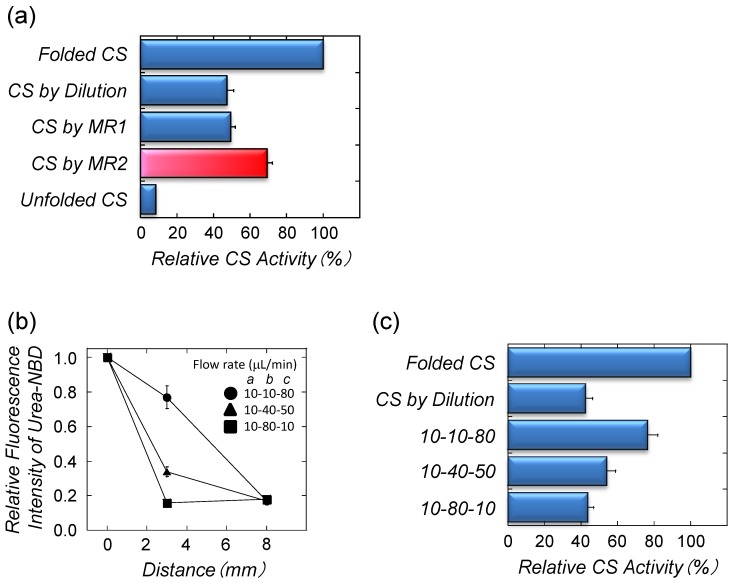
Citrate synthase (CS) refolding by microfluidic chips [15]. (**a**) The recovered enzymatic activities of CS using different refolding approaches. Flow rates for MR1: channel *a* (denatured CS), 10 μL/min; and channel *b* (buffer), 90 μL/min. Flow rates for MR2: channel *a* (denatured CS), 10 μL/min; channel *b* (buffer), 10 μL/min; and channel *c* (buffer), 80 μL/min. Folded CS was prepared by dialysis. Unfolded CS was assayed in 2.5 M urea. The diluted sample was directly diluted by buffer in a test tube; (**b**) The effect of the different flow rates of the diluting buffers (channels *b* and *c*) on CS refolding. Relative fluorescence intensities of NBD in the urea stream at the junctions in MR2; (**c**) The recovered enzymatic activities of refolded CS in MR2. Flow rates of channels *a*, *b*, and *c* (μL/min) are shown in the graph.

The refolded CS prepared using MR1 with one junction showed similar recovered enzymatic activity (<50%) to that of the diluted sample, and less helical structure than the folded CS sample, suggesting that rapid diffusion of urea from the denatured CS leads to misfolding. In contrast, the refolded CS sample prepared using MR2 with two junctions showed a more helical structure than CS prepared by dilution and MR1 [15]. The recovered activity was also enhanced in CS prepared using MR2 compared with the batch and MR1 samples (Figure 5a). These results indicate that denatured CS was more efficiently refolded using MR2 compared with protein refolding by dilution and MR1. The recovered enzymatic activity of CS by MR2 (>70%) is a similar value to protein refolding by the artificial chaperone-assisted system, which is an efficient technique to recover active proteins from denatured forms [47,48].

The laminar flow conditions allowed control of the urea distribution in the microchannel of MR2 (Figure 4c). Thus, the effect of different flow rate ratios of the refolding buffers on the CS refolding yield was studied. In this experiment, the flow rate of denatured CS was maintained at 10 μL/min (channel *a*) and the total flow rate of the refolding buffer was 90 μL/min (channels *b* and *c*) with different flow rate ratios (flow rate in channel *b*: flow rate in channel *c*). Changes of the flow rate ratio of the buffers showed different decreasing trends of the urea concentrations (Figure 5b). A low flow rate of the buffer in channel *b* (10-10-80 in Figure 4 and Figure 5) resulted in a slow decrease in the urea concentration, leading to highly recovered enzymatic activities (Figure 5c). 

The refolding of proteins using microfluidic chips with multiple junctions was achieved within a short period of time at room temperature. The estimated throughput of our CS refolding method, using MR2, was 150 μg/h. This value is one order of magnitude higher than the throughput of the artificial chaperone-assisted system (9−14 μg/h in Ref. 49). These results suggest that a gradual decrease of the denaturant concentration in the microchannel can provide the equilibrium between unfolding and refolding (native conformation) and not misfolding and/or aggregation. 

### 4.3. Refolding of Recombinant Protein from Inclusion Body by Microfluidic Chips

The designed chips have been evaluated for their refolding performance on recombinant protein from inclusion bodies. *ζ*-Associated protein 70 kDa (ZAP-70) is a tyrosine kinase [64]. Because overexpressed ZAP-70 in the bacterial expression system makes inclusion bodies, it is usually expressed in the mammalian or insect expression system [64,65]. However, these expression systems are expensive compared with the *E. coli* system, and the recovered yield of the protein is generally low. 

In the refolding experiments, the urea-denatured ZAP-70 protein kinase domain (mouse residue 337–597), which was purified from *E. coli* inclusion bodies, was applied to the microfluidic chips to evaluate protein refolding. The circular dichroism (CD) spectrum of refolded ZAP-70 using MR1 showed a similar spectrum to the batch sample by dilution. In contrast, the CD spectrum of ZAP-70 prepared using MR2 was similar to that of folded ZAP-70 prepared by step-wise dialysis over two days. The estimated helical content of ZAP-70 using MR2 was higher than that of ZAP-70 prepared using MR1 and dilution [15]. 

These results indicate that microfluidic chips may provide miniaturized tools for rapid and efficient recovery of active proteins from inclusion bodies.

## 5. Conclusions

Recovering biologically active proteins at low cost is the important goal in protein refolding from bacterial inclusion bodies, not only for analysis of the protein structure and function [66], but also for the development of therapeutic drugs and industrial processes [1,2]. Refolding is the change of the protein conformation from unfolded to folded, and is dependent on the denaturant concentration. As rapid decreases in denaturant concentration lead to misfolding and/or aggregation [17,19], a gradual decrease in denaturant concentration within a short period of time may lead to efficient protein refolding. In this review, we introduced the refolding methods using laminar flow in microfluidic chips and chemical additives. The estimated throughput of the protein refolding method using microfluidic chips with multiple junctions is 100−200 μg/h [15], suggest that this refolding method may serve as miniaturized tool for laboratory-scale protein recovery. In contrast, although the refolding method using chemical additives may be suitable for industrial purposes, identifying the refolding buffer conditions, such as pH and ionic strength, and the selection of suitable chemical additives is still a major bottleneck. Thus, an automated robotic platform has also been studied to develop a screening system for the dilution refolding process [67]. 

Recently, an artificial chaperone-assisted refolding method was combined with microfluidic technology [58]. Although protein refolding by dilution in a microchannel led to the formation of protein aggregates because of the rapid removal of denaturant, the artificial chaperone molecule effectively suppressed these protein aggregates at the mixing point of the denatured protein and the diluting buffer [58]. Another combination technique with a molecular chaperone and polyethylene glycol has been reported [68]. These studies suggest that combination of the different technologies is a promising approach and could improve the results of current protein refolding techniques. In addition, studies on the interaction between proteins and between proteins and chemical reagents, such as denaturants and additives, may help us to understand the molecular mechanisms in detail and could progress refolding technology. 
